# Nanofibers of Cellulose and Its Derivatives Fabricated Using Direct Electrospinning

**DOI:** 10.3390/molecules20059139

**Published:** 2015-05-19

**Authors:** Kousaku Ohkawa

**Affiliations:** Division of Biological and Medical Fibers and Institute for Fiber Engineering (IFES), Interdisciplinary Cluster for Cutting Edge Research (ICCER), Shinshu University, Tokida 3-15-1, Ueda 386-8567, Nagano Prefecture, Japan; E-Mail: kohkawa@shinshu-u.ac.jp; Tel.: +81-268-215573; Fax: +81-268-215571.

**Keywords:** electrospinning, electrospun non-woven fabrics, cellulose, chitosan, functionalization

## Abstract

A short review with 49 references describes the electrospinninng (ES) process for polysaccharides, cellulose and chitosan, and their derivatives, including cellulose acetate and hydroxypropyl cellulose. A majority of applied studies adopted a two step-process, in which the cellulose acetate was used for the first ES process, followed by acetyl group removal to regenerate cellulose thin fibers. The electrospun nonwoven fabrics (ESNW) of regenerated cellulose can be modified by introduction of aldehyde groups by oxidative cleavage of vicinal diols using periodates, and these aldehyde groups serve as acceptors of foreign substances, with various chemical/biological functions, to be immobilized on the fiber surfaces in the ESNW matrices. Direct electrospinning of cellulose from trifluroacetic acid solution was also developed and the applied studies were summarized to conclude the current trends of interests in the ES and related technologies.

## 1. Introduction

Since a published patent [1], considered the historical origin of the electrospinning (ES) technology, described a polymer material, cellulose acetate (CA), the nanofiber fabrication technique and cellulose-related materials have jointly studied for a long time. The principle, theories, and experimental setup variations for ES techniques can be found in other reviews from the early 2000s [2,3,4]. The sheet-like materials fabricated via the ES process, with micro-scale structures involving a non-woven network of very thin fibers, are called “Electrospun Non-Woven Fabrics (ESNWs)”. The original papers and review articles with various research purposes/motivations and studies have employed different terms for the electrospun fabrics, for instance, electrospun- (or nanofiber-) mats, -sheets, -non-woven films, -porous membranes, *etc.*, thus in this article, the author will use the term ‘ESNWs’ to avoid any inconsistencies in the definition of the electrospun fabrics.

The crystalline polymers, including the naturally-occurring polysaccharides, cellulose and chitin, and chitosan are also much less soluble, and their available solvents are highly dielectric and not very volatile in many cases, and thus the known solvents of cellulose and chitosan are not routinely used for the ES process. These facts established an important objective in fine fiber technologies to determine the favorable solvents for the ES process of cellulose and chitosan. This article reviews the topics concerning the ES process of cellulose, chitin and their derivatives found in recent research publications.

## 2. ES Processes of Cellulose and Cellulose-Derivatives

### 2.1. Cellulose-Derivatives

Table 1 summarizes the list of publications, mainly from 2010 to 2012, and the corresponding content previews of their topics, including material polymers, cellulose and derivatives, ES solvents, average diameters, and purpose of the research studies. In a strict sense, the terms ‘nanofibers’ and ‘nano-scaled fibers’ define those having average diameters less than 100 nm [3], however, most publications in Table 1 have applied the term ‘nanofibers’ for their fabrics, even in the cases where the average diameters were 200–800 nm (submicro- or micro-scaled). In addition to this inconsistency, several research studies in Table 1 include the phrase ‘electrospun cellulose nanofiber’ in their title when, in fact, the starting material used was CA, so that the readers should pay attention so as to not confuse the starting materials, the processing solvents, and the presence or absence of any pre- or post-processing of the ESNWs.

In many of these research studies that employed CA as the starting material, the post-spinning deacetylation of the resulting CA-ESNWs using an alkaline solution produces cellulose-ESNWs. The resulting cellulose-ESNWs in these research studies are described as ‘regenerated cellulose (RC)’, but since the term ‘regenerated cellulose fibers’ has already been used for several cellulose-based industrial fiber products, and CA itself has been established in commercial products, the meanings of the term, ‘regenerated’ in ‘cellulose-ESNW’ and in ‘cellulose fibers’ are strictly different in a sense of use. A two-step method, which involves the conversion of CA-ESNW to RC-ESNW via a post-spinning deacetylation, has been applied by many researchers. The reason is as follows: as described in the Introduction, favorable solvents for the ES process of cellulose have not been easily discovered/developed, while the ES processing of CA, of which the acetyl group content is approximately 40% and the molecular weights of 30,000 to 60,000, has already been established using mixed solvents of acetone and aprotic solvents, for instance, dimethyl formamide to dimethyl acetamide.

**Table 1:** List of publications on cellulose- and cellulose-derivative-ESNWs (publication year, *ca.* 2010 to 2012).

Matrix Polymers	Solvents (Concentrations)	Fiber Diameters in Averages (nm)	Post-Spun Treatments and Focus of Research	References
CA ^(1)^	Acetone/DW = 5:1 (8%)	300–500	Deacetylation using 50 mM NaOH for conversion to RC-ESNWs; Detection of metal ions	[5]
CA: PVA	CA, Acetone/DMF = 2:1 (17 wt %) PVA, DW (10 or 15 wt %)	CA, 740 nm PVA, 340 or 710 nm	Independent nozzles for CA and PVA solutions to prepare composite ESNWs; Deacetylation using 50 mM NaOH for conversion to RC-ESNWs; Evaluation of hydrophilic properties before and after deacetylation.	[6]
CAP ^(2)^ (Mw ~60,000 g/mol)	Aceton/DMF = 3: 1 (25 wt/v %)	500–800	Sustainable release of low molecular weight substances having anti-virus activity, suppression of infective activity of the virus, and so on.	[7]
CA (Mw, 25,000)	Acetone/DMF = 1: 4 (14 wt %)	170 ± 40 nm	Antibacterial activity expression by fabrication of nano-composite with ZnO.	[8]
CA (Dp, 200; DA, 2.5)/RSF ^(3)^ = 1:9	Formic acid (12 wt %)	142 nm ± 32%	Changes in mechanical properties of ESNWs though composite fabrication and metal ion adsorption.	[9,10]
PMIA ^(4)^/CA (1:2) or cellulose (6:1)	PMIA, 8 wt % LiCl/DMAc (15%) Cellulose, swelled in 8 wt % LiCl/DMAc (10%) CA, MDAc (15 wt %)	PMIA/CA, 250–300 nm PMIA/cellulose, 250–350 nm	Development of ES process and reinforcement of mechanical properties of ESNWs.	[11]
CA (Mw, 61,000 g/mol, acetylation 40%)	Acetone/DMAc = 2:1 (20 wt %)	750 nm (500–1500 nm)	Graft polymerization of methacrylic acid on CA and evaluation meal ion adsorption.	[12]
CA (Mw, 30,000, actylation 39.8%)	Acetone/DMAc = 2:1 (16 wt/v %)	385 nm	Deacetylation using 0.3 M NaOH, then condensation of Oxolane-2,5-dione (succinic anhydride), then evaluation of metal ion adsorption.	[13]
CA (Mw, 30,000 g/mol, acetylation 39.8%)/PEO ^(5)^ (Mn, 300,000 g/mol)	CA, 99.9% AcOH (20 wt/v %) PEO 90% EtOH (3 wt/v %)	CA soln/POE soln = 99.6/0.4–91.9, 950–1170 nm	Composite ESNWs with ZnO nano-particles, changes in mechanical properties, and application for food wrapping technology.	[14]
CA (Mw, 30,000 Da, actylation 39.7 wt %)	Acetone/DMAc = 2:1 v/v% (17 w/w %)	701–1057 nm	Development of ES process, and *in vitro* evaluations on sustained release of gallic acid (3,4,5-trihydroxy benzoic acid)	[15,16]
Cellulose (Dp, 1100)	[EMIM] [OAc] ^(6)^ (1.75 wt %) *	1000–2500 nm **	A fundamental study on fabrication of CB^(7)^ -binding ESNWs for application as affinity ligand carrier.	[17]
Cellulose (Dp, 1100)/chitosan (800 cP) or/PMMA^(8)^ (120 kDa)	[EMIM] [OAc] (Cellulose, 2 w/w%)/chitosan, 0.4 w/w %) [EMIM] [OAc]/DMSO/TritonX100 (cellulose, 2.25 w/w %/PMMA 0.5 w/w %) *	Cellulose/chitosan, 150–450 nm Cellulose/PMMA, 150–350 nm	Partial oxidative cleavage using periodates to generate aldehyde groups to bind Lysostaohin in a fundamental study for application as antibacterial wound dressing materials.	[18]
Cellulose/Cellulose nanocrystal CNC)	Cellulose, NMMO^(9)^/DW = 4: 1 wt (1.5 wt %), Shell solution CNC, DMSO (0.42–3.28 wt %), core suspension	~100 nm	Development of ES process using layered nozzles having shell (cellulose in NMMO) and core (CNC dispersion in DMSO); Relationship between crystalinities of CNC and modulus of ESNWs.	[19,20]
Cellulose (cotton linter, Dp, 12,000)	8.5 wt % LiCl/DMAc (1.0–1.35 wt %)	80–100 nm	Immersion in water to revome residual LiCl and development of ES process for high molecular weight cellulose preparation.	[21]
polyquaternium-4 cellulose (PQ-4) (N, 1.5%–2.3%)/PVA (Dp, 1750 ± 50, DA, 98%) = 1:3, 1:2, 1:1	PQ-4, DW (2–8 wt %) PVA, DW (1, 10 wt %) PQ-4/PVA, DW (7 wt %)	PQ-4, no-spun PQ-4/PVA 1:3, 202–217 (±38–59) 1:2, 157–246 (±46–66) 1:3, 211–249 (±60–71)	Antibacterial activity expressed by blend with PQ-4.	[22]
CA (acetylation, 29.6%)	Acetone/DMAc = 3:2 (15 wt %)	200 nm	Deacetylation using 0.5M KOH/EtOH solution, then partial oxidative cleavage with periodate to generate aldehyde groups to immobilize an enzyme, lipase, and evaluation of immobilized enzyme activity.	[23]
CA	TFE ^(10)^ (150 g/L)	200 nm–3000 nm	Deacetylation using 0.1 N NaOH in EtOH/DW = 1:4, then partial oxidative cleavage using sodium iodate to generate aldehyde groups to bind 2-aminoethyl sulfate and evaluation of scaffolding properties for cell culture.	[24]
CA (Mw, 30,000)	DCM ^(11)^/Acetone = 1:1–3:1 (v:v) (5.0, 7.5, 10 w.v %)	DCM/Acetone = 1:1, CA, 10 wt %, 300–1000 2:1, CA, 7.5 wt %, 750–1500 3:1, CA 5 wt %, 1500–3500	Development of CA fine fibers having porous structures.	[25]
CA (Mw, 61,000, acetylation 40%)	AcCN ^(12)^/0–30w/w %EtOH (15–21 w/w %)	Sub-micrometric fibers	Vacuum deposition of Al, Cu, and Ag onto CA-ESNWs and evaluation of electrochemical properties for application as bio-battery.	[26]
CA (Mw, 30,000 g/mol)/PBA ^(13)^ (610,000 g/mol) = 90:10, 80:20, 70:30 (w/w)	Acetone//DMF = 8:2 (w/w)/0.2 wt % isocyanate	CA/PBA = 90:10, 510 80:20, 1700 70:30, 3980	Curing using isocyanate and effect of the treatment on the fin fiber morphologies of ESNWs.	[27]
CA (Mw, ~30 kDa, acetylation 39.7 wt %)/EA ^(14)^ (14–76 kDa) = 91:9, 77:23, 66:34 (w/w)	AcOH/formic acid/DW/tween40 (CA/EA = 91:9, 19.25wt%; 77:23, 18.25 wt %, 66:34, 17.25 wt %)	CA/EA = 91:9, 242 ± 32 77:23, 384 ± 54 66:34, 410 ± 38	A fundamental study on development of eatable fine fiber ESNWs for application as *in vivo* sustainable release mateials.	[28]
CA (d, 1.184 g/cm^3^; Tm, 280 °C)/PAN ^(15)^ (d, 1.184 g/cm^3^, Tm, 317 °C)/MWCNT ^(16)^ (d, 1.2–1.7 g/cm^3^) =	CA, Acetone/DMAc =1:2 (15 wt %/10 wt %), core soln. PAN/MWCNT, DMF (10 wt %/35 wt %), shell susp.	150 (core, ~60; shell, ~50)	Development of ES process for highly loaded CNT composite ESNWs, and evaluation of thermostabilities of ESNWs for application of electronic devises.	[29]
CA	Acetone/BzOH ^(17)^ = 2:1 (14 wt %) MEK ^(18)^/BzOH = 4:1 (14 wt %) Acetone/DMSO =2:1 (18 wt %), others	3410 ± 1780 2030 ± 660 650 ± 130	A fundamental investigation of processing condition, including solubilization parameters of solutes and solvents, viscosity, and correlation with the fibrous structures.	[30]
CA (Mn, 30,000, acetylation 39.8)	Acetone/DMF/DW = 85%/10%/5% (v/v/v) (17 v/v %)	500 (100–1000)	Development of Quillaja sapnin-loaded ESNWs then deacetylatioin using 50mM NaOH aq/EtOH and evaluation of sub-component yields in RC-ESNW and antimycotic activities.	[31]
CA (Mn, 30,000, acetylation 39.8%, DS, 2.46)/POSS ^(19)^	Acetone/DMAc = 2:1 (CA, 15 wt %/) POSS, 3 or 5 wt %CA	POSS3 wt %, 262 ± 59 POSS5 wt %, 269 ± 50	Development of organic/inorganic composite ESNWs having nano-scaled dispersion of the inorganic sub-components.	[32]

^(1)^ cellulose acetate, ^(2)^ cellulose acetate phthalate, ^(3)^ Regenerated silk fibroin, ^(4)^ poly(m-phenyleneisophthalamide), ^(5)^ poly(ethylene oxide), ^(6)^ 1-ethyl-3-methylimidazolium acetate, ^(7)^ Cibacron Blue F3GA, ^(8)^ poly(methyl methacrylate), ^(9)^ N-methylmorpholine oxide, ^(10)^ trifluoroethanol, ^(11)^ dichloromethain, ^(12)^ acetonitrile:, ^(13)^ poly(butyl acrylate), ^(14)^ egg albmen powder, ^(15)^ poly(acrylonitrile), ^(16)^ multiwalled carbon nanotubes, ^(17)^ benzyl alcohol, ^(18)^ methyl ethyl ketone, ^(19)^ polyhedral oligomeric silsesquioxanes, co-electrospining with two independent nozzles/mixed solution; *: Collectors were immersed in water or water:EtOH = 1: 1 bath for coagulation; **: No description in original article, estimated from the corresponding figures. Units of concentration are as used in the references.

Since the first patents by Formhals in 1937 [1,33], the chemical conversion of CA-ESNW to RC-ESNW was only documented by Hsieh in 2002 [34], which means that it took almost 35 years to realize the successful fabrication of RC-ESNWs. There are now multiple ways to functionalize RC-ESNWs by a chemical reaction to generate aldehyde groups on the surface of the fine RC-ESNW fibers via oxidative cleavage of vicinal diols using an aqueous periodate solution, and the aldehyde groups work as acceptors of foreign ligands, including biologically active substances, enzymes, or affinity-targeted molecules, which are discussed in Section 3.1. Cellulose nitrates can be also fabricated as ESNWs [35].

### 2.2. ‘Direct’ ES Process of Cellulose Solutions

In 2004, Ohkawa *et al*. published a research study on the ES of chitosan solution [36], the solute polymers of which have viscosity average molecular weights ranging from *ca.* 200,000 to 1,800,000, and then in 2006, the fabrication of chitosan-ESNW, of which the average diameters of single fine fibers were less than 100 nm, was realized [37], meaning that the first time “nanofiber chitosan-ESNW” came to the materials science of ES processing. Various combinations of organic acids were then examined as ES solvents, and among them, trifluoroacetic acid (TFA) was found to be the most favorable one for the ES-processing of chitosan [38]. Later in 2009, the authors found that cellulose-ESNW in which the average diameters of single fibers ranged from 100 nm to submicrometers, was fabricated when the TFA solvent was applied to the ES process of cellulose preparations from pulp and cotton [39]. This result has newly allowed the direct ES processing of cellulose preparations, without involving a cellulose derivative such as CA, and the authors called this one-step process without any pre- and post-spinning treatment ‘direct’ ES (dES).

Other research studies classified as dES employ aqueous *N*-methylmorpholine-*N*-oxide (NMMO aq.), which is a well-known solvent for regenerated cellulose fiber in industrial scale production, and a spruce cellulose preparation (Dp, 700–800) was fabricated as cellulose-ESNW (average diameter, 200–400 nm) in the presence or absence of the mercelization [40]. *N*-Alkylinidazolium-derivative ionic liquids (IL) have also been examined as processing solvents for the dES of cellulose [41,42] or a composite of cellulose and chitosan [43]. In the cases of dES processes using NMMO aq. and IL, the evaporation of solvent molecules might not occur, hence the fiber collector was a metallic plate immersed in water or alcohol. *N*,*N*-Dimethylacetamide containing *ca.* 8.0 wt % LiCl was also reported as a dES solvent in cellulose preparations, and after evaporation of the solvent, the remaining LiCl in the fiber matrix on the collector was removed by rinsing with water [11].

**Figure 1:**
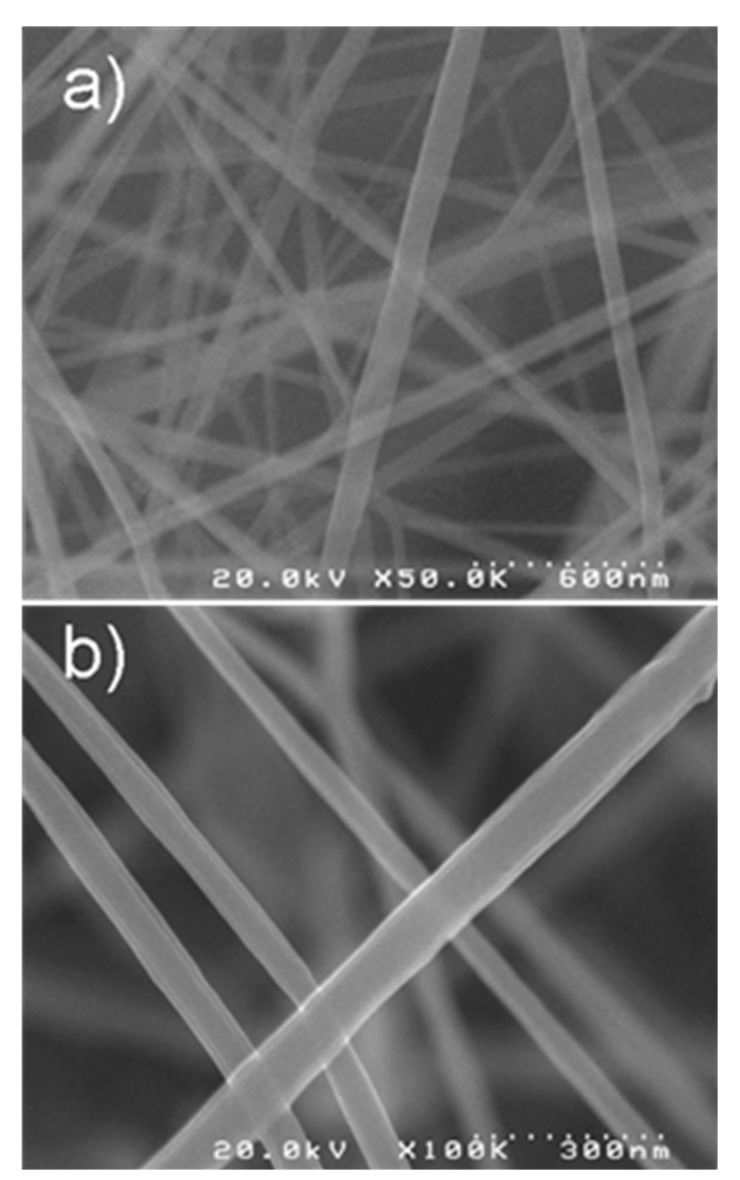
(**a**) Pulp cellulose-ESNW (scale bar, 600 nm); (**b**) Cotton cellulose-ESNW (scale bar, 300 nm). Reproduced from [44] with permission of SAGE Publications, Ltd.

Other solvents for the ES process of cellulose can be found in several patent documents, but most of them describe solvents which are frequently used in the production of regenerated cellulose fiber. In several cases, the processes require pre- or post-ES spinning treatments. The dES of cellulose using perfluoro-organic acids [44] typically yields very fine fibers with the smallest diameter of 60–75 nm without any pre- or post-spinning treatment (Figure 1).

## 3. Applied Research Studies for the Functionalization of Cellulose- or Cellulose-Composite-ESNWs

### 3.1. Chemical Modification of Cellulose-ESNWs

A review [4] describes in detail the applied studies of the fine fibers of organic polymers fabricated by ES processes. When compared to other fabrication methods, e.g., melt-browns, the lower production performance of the ES process is considered a problem, however, during the last decade, the problem has been solved with the development of suitable processing machinery for the large-scale fabrication of ESNWs, and hence, the scope and criteria of the applied studies of ESNWs has become wider and more varied.

One of the more remarkable functionalization strategies is chemical modification of the RC-ESNWs. Among the research studies listed in Table 1, periodates were used for the treatment of RC-ESNWs for generating aldehyde groups via oxidative cleavage of vicinal diols in the deacetylated glucopyranoside units. The aldehyde groups can immobilize various kinds of ligands, and thus the applied studies of RC-ESNWs encompass the scopes of: (i) scaffolds for cell culture; (ii) absorbents of heavy metal ions for healthcare or environmental technology; (iii) materials for the separation of conjugated and affinity linked bilogical substances; (iv) immobilized catalysts including enzymes; and (v) drug-releasing materials having antibacterial activities. A common feature of these objectives is utilization of the large surface area characteristic of the fine fiber ESNWs, and in some cases, a nano-scaled dispersion of the composite particles also having nanoscale sizes, while the biological and environmental compatibilities of cellulose are taken for granted.

**Figure 2:**
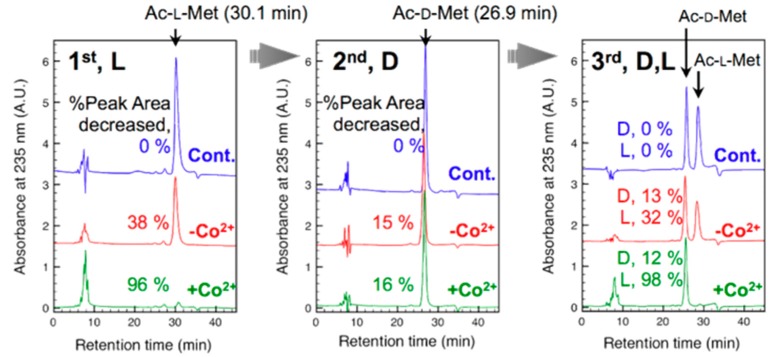
Chemical modification of HPC-ESNWs for preparation of a cationic matrix.

Ohkawa *et al*., described the conjugation of ligands via an electrostatic interaction between hydroxypropyl cellulose (HPC)-ESNWs. The surfaces of the fine fibers in the HPC-ESNW matrices are chemically modified using diasocyanate cross-linkers (Figure 2) [45]. The excess amount of the cross-linkers in the treatments plays two roles; one is just for cross-linking, which renders the HPC-ESNWs insoluble in most solvents, and the other one is for leaving unreacted NCO groups, which can be utilized both as: (a) a direct acceptor of any substance molecules having nucleophilic moieties, and (b) if *N*,*N*-diethylethylene diamine is used for the linkage via (a), strongly cationic (diethylaminoethyl—DEAE) groups can be introduced on the surfaces of the HPC fine fibers. These ideas have been derived from the method to prepare the DEAE-cellulose for immobilizing enzymes based on the original studied by Chibata *et al.* [46].

Aminocylase-I from porcine pancreas catalyzes the hydrolytic reaction of *N^α^*-acetyl-l-amino acids to yield the free l-amino acid, and the enzyme is highly active for l-Met and l-Leu.

**Figure 3:**
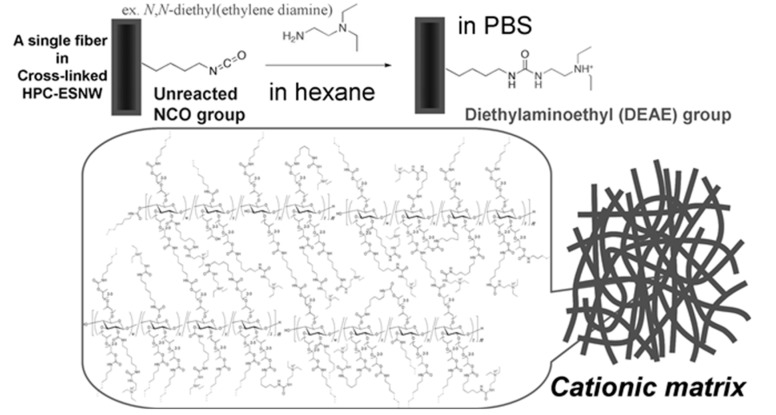
Chirality-specific hydrolysis of Ac-l-Met by aminoacylase-I-immobilized HPC-ESNWs. More details for experiments are described in reference [45]. Left, most all of Ac-L-Met fed were hydrolyzed; Middle, No hydrolysis was observed for Ac-d-Met except for decreases in peak area due to absorption; Right, Only Ac-l-Met was hydrolyzed in a racemic mixture. Co^2+^ is a cofactor for enhancing the enzymatic activity.

When aminoacylase-I was immobilized on the DEAE-HCP-ESNWs, the immobilized aminoacylase-I exhibited a deacetylation catalysis with an l-specific chiral recognition towards l-Met, as seen for the free enzyme, and in repetitive cycles of use, the initial activity and L-specificity were mostly retained (Figure 3).

### 3.2. Cellulose-Composite-ESNWs

Another direction for applied research studies of the cellulose-ESNWs is to prepare cellulose-composite-ESNWs, and in these cases, cellulose derivatives are the major components, which carry subcomponents for functionalization, for instance, synthetic and natural polymeric materials, low molecular weight organic or inorganic substances, and carbon nanotubes for their applied studies from the viewpoints of electronic devices, hydrophobic (superabsorbent) polymers for healthcare and medical uses, antibacterial surface processing, reinforced composites, and sustained release materials. Most of the subcomponents were in the forms of nano- or submicro-scaled particles or whiskers and blended with the cellulose derivatives in the pre-spun solutions. A common feature in these studies is utilization of the chemical, mechanical, or electric properties of the subcomponents with a large surface area and nano- or micro-scaled dispersions/orientations.

When the shapes of the subcomponents are small sized whiskers or carbon nanotubes, a concentric spinneret is often employed to fabricate core-shell composite fine fibers [19,20,29]. References about both cellulose derivatives and coaxial electrospinning enable: (i) zero-order drug release cellulose acetate nanofibers; (ii) the preparation of high-quality ketoprofen-loaded cellulose acetate nanofibers; and (iii) electrospun biphasic drug release using polyvinylpyrrolidone/ethyl cellulose core/sheath nanofibers. Another fabrication method for composite ESNWs of polymers and cellulose-derivatives involves multi-nozzle jet emission, in which different kinds of polymer and cellulose derivative solutions were emitted from each of the independent nozzles by an applied voltage [6]. Among the research studies listed in Table 1, almost no dES methods were found, especially for the cases that cellulose itself (not its derivatives) is used as a major component. The reason for that is limitation in the choice of the ES solvent, whereby most of the available solvents, for example TFA, are highly acidic and strongly dielectric, and these properties will affect the chemical or electrical properties of the subcomponents. On the other hand, in the case of the CA-ESNWs, available solvents for the ES process are neutral, so that the stability of the subcomponents is not chemically damaged. The majority of researchers may be concerned with the preparation of the cellulose-composite-ESNWs being difficult, especially for biological subcomponents, including enzymes.

**Figure 4:**
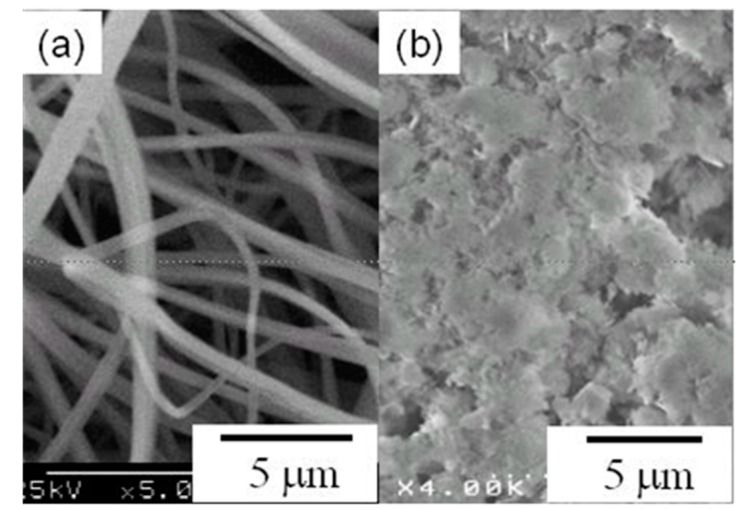
(**a**) Scanning electron microscopy of a Cellulose-copoly [Ser(PO_3_H_2_)*^x^*, Asp*^y^*]-Composite-ESNW (*x*:*y* = 75:25) (1.0 wt % towards cellulose); (**b**) Morphology of hydroxyapatite after *in vitro* calcium phosphate deposition, which entirely covers the ESNW matrix.

Ohkawa *et al*. have continued their applied studies on cellulose-composite-ESNWs using sub-components which are relatively stable, even in neat TFA solutions. Figure 4 is an example study for this approach, and the cellulose-ESNW subcomponent is a synthetic polypeptide, copoly[Ser(PO_3_H_2_)*^x^*, Asp*^y^*] (*x*:*y* = 100:0, 75:25, 50:50, 25:75), on which serine residues are fully phosphorylated. The weight ratio of copoly[Ser(PO_3_H_2_)*^x^*, Asp*^y^*] towards cellulose was 1.0–1.2 wt % in a TFA solution, and then after the ES process, the products, cellulose-copoly[Ser(PO_3_H_2_)*^x^*, Asp*^y^*]-composite-ESNW, was observed using a scanning electron microscope (Figure 4) [47]. The chemical structure of copoly[Ser(PO_3_H_2_)*^x^*, Asp*^y^*] was designed on the basis of osteogenic proteins, of which their role is to induce the hydroxyapatite deposition to make hard tissues, teeth and bone. Since the synthesis route of the copolypeptide involves the final deprotection step in TFA, the ES solvent of the cellulose-copoly[Ser(PO_3_H_2_)*^x^*, Asp*^y^*]-composite-ESNW does not affect the chemical structure of the subcomponent. A series of *in vitro* experimental results indicated that the composite ESNWs were able to induce hydroxyapatite deposition, which is associated with the crystalline polymorph transition similar to those occurred *in vivo*, and potential application of the cellulose-copoly[Ser(PO_3_H_2_)*^x^*, Asp*^y^*]-composite-ESNWs is, hence, applied as the scaffolding materials for hard tissue engineering. As described above, reasonable design of the subcomponents and their chemical structures, which are derived from biological components, enables biomedical studies by the dES process and fabrication of cellulose-based-composite ESNWs [48,49].

## 4. On a Specific Issue of Cellulose-ESNWs Prepared from TFA Solutions

There is a specific issue when considering the potential applications of cellulose-ESNWs prepared via the dES process using TFA solutions. The term ‘cellulose nanofiber’ frequently appears in research articles, reviews and book chapters/sections, which interests are usually the I-type crystallites of cellulose, often referred to as ‘cellulose nano-crystals (CNCs)’ or ‘cellulose nano-whiskers (CNWs)’. As already described in references [44,49], the cellulose-ESNWs prepared via dES from perfluoro-organic acids do not exhibit any of significant diffraction characteristic of I-III-type crystallites, which suggests that the cellulose molecules in the ESNW matrices adopt an amorphous state.

The fact mentioned above implies that the feature of the high modulus, I-type crystallites, is absent in the cellulose-ESNWs fabricated via the dES process. The directions of material design policies between the high modulus composites using CNCs or CNWs and the cellulose-nanofiber-ESNW via the dES process have to be distinguished on the basis of their principles. The latter cannot encompass the aim to develop a high modulus composite design, while the amorphous structures of cellulose matrices in the ESNWs inspire another policy to express the inclusion or intercalation properties of the cellulose-ESNWs, for instance, sustainable drug-release or the entrapment of heavy metal ions. The difference between the utilizations of the CNCs or CNWs and the electrospun cellulose nanofibers is remarkable, although both policies are classified in the developmental approach of ‘cellulose nanofibers’.

Recently, a characteristic trial was performed to combine both features described above, and a reference [20] in Table 1 describes a fabrication method for cellulose-CNC-composite-ESNWs in which a double layered nozzle was employed to install the shell layer of a cellulose solution in NMMO aq. and the core layer of a CNC dispersion in dimethyl sulfoxide (DMSO). This combined approach will require several precise regulations, including the suppression of the CNC aggregation and the sufficient retention of the CNC weight fraction for significant reinforcement of the cellulose-CNC-composite nanofibers regarding their mechanical properties, while these conflicting features will not easily coincide, thus ensuring the preferable processing parameters, for instance, viscosity of the pre-spun dopes. The fundamental studies on the improved design of nozzles and on the reproducibility of the reinforcement effect for the modulus of every single nanofiber still remain to be investigated, as well as the combined approach, cellulose-CNC-composite-ESNWs, is an important subject in researchers interests to be counted as a technical work-in-progress.

## 5. Concluding Remarks

The trend in both fundamental and applied research on cellulose- and the cellulose-derivative ESNWs clearly indicates that this approach involves multidisciplinary science, which encompasses agriculture, engineering, and medicine. Including the related β(1,4)-type polysaccharides, chitin and chitosan, the scope for the practical applications of the ESNWs fabricated using natural polysaccharides and their derivatives will be extended with the increasing number of research publications, which have been growing in number remarkably since the latter half of the 1990s.

The relationships between basic polymer science and applied studies in ES technologies are complimentary to each other. Polymer science and the chemistry of cellulose have very long histories for both research and industries, thus, since the fabrication mechanism of nanofibers and ESNWs can also be understood and discussed, once the first cellulose-ESNW was developed and reported, the cellulose-ESNW technology immediately inspired researchers to hybridize it with the knowledge already accumulated in cellulose science. As a result, a variety of ideas summarized in Table 1 was developed within a very short period. There is a few number of examples in industrial applications of the cellulose- and cellulose-derivative ESNWs, however, due to the background mentioned above, the cellulosic ESNWs might have the most promising potential for industrialization. The recent progress in electrospinning technologies of chitin and chitosan [50,51,52] will also inspire/expand the potential applications of chemically modified (amino-functionalized) cellulose nanofibers [53,54,55].
